# A pan-cancer analysis of the oncogenic role of branched-chain aminotransferase 1 (BCAT1) in human tumors

**DOI:** 10.1097/MD.0000000000050004

**Published:** 2026-07-31

**Authors:** Binbin Wang, Yize Wei, Mingjie Dong, Congrong Gao, Hui Yue, Ming Sun

**Affiliations:** a Department of Thoracic and Cardiac Surgery, Hefei Hospital Affiliated to Anhui Medical University, The Second People’s Hospital of Hefei, Hefei, Anhui Province, China.

**Keywords:** BCAT1, cancer, prognosis

## Abstract

Based on emerging evidence implicating branched-chain aminotransferase 1 (BCAT1) in tumorigenesis from animal and cellular studies, this study aimed to systematically evaluate its oncogenic role through a pan-cancer analysis to address the lack of comprehensive pan-cancer investigations in the literature. Using datasets from The Cancer Genome Atlas and the Genotype-Tissue Expression project, we analyzed BCAT1 expression, prognosis, genetic alterations, immune infiltration, and functional enrichment across 33 cancer types. The primary endpoints were overall survival (OS) and disease-free survival (DFS). Statistical significance was primarily defined as *P* < .05. For analyses involving multiple correlations across cancer and immune cell types, the false discovery rate < 0.05 was applied. BCAT1 was significantly upregulated in most malignancies, and high expression was associated with worse OS and DFS in multiple cancer types. BCAT1 expression correlated with reduced CD8+ T cell infiltration and increased cancer-associated fibroblast accumulation across several tumors. Enrichment analyses suggested BCAT1 involvement in RNA metabolism and intracellular protein processing. BCAT1 was significantly upregulated in most malignancies, and high expression was associated with worse OS and DFS in multiple cancer types, including adrenocortical carcinoma (hazard ratio = 6.3) and lower-grade glioma (hazard ratio = 2.6) (all log-rank *P* < .05), highlighting its potential as a pan-cancer biomarker and therapeutic target. The present findings are associative and require further experimental and external validation.

## 1. Introduction

The capacity of cancer cells to proliferate and maintain their growth is limited. The tumorigenesis process relies on amino acids, which are the building blocks of protein synthesis, as well as sources of energy and metabolites obtained from the tumor microenvironment.^[1]^ Given the intricacy of carcinogenesis, it is crucial to carry out a pan-cancer expression analysis of the genes involved in nutrient uptake and assess their significance for clinical prognosis.

Many cancer types provide cellular energy and metabolites for anabolic processes by overexpressing enzymes that degrade amino acids.^[2]^ One class of amino acids whose metabolism has been linked to particular cancer characteristics is the branched-chain amino acids (BCAAs).^[3]^ When BCAAs are ingested by cells, they are 1st decomposed by the BCAT family. In this family, branched-chain aminotransferase 1 (BCAT1) BCAT2 are involved in the decomposition of branched amino acids. Since then, pertinent data have shown that BCAT1 exhibits aberrant behavior in various malignancies.^[4–6]^ As a result, BCAT1 is thought to be more closely linked to tumor growth than BCAT2. Mounting evidence suggests that BCAT1 is overexpressed in a variety of tumor tissues.^[7,8]^ However, based on large clinical data, there is no evidence of a pan-cancerassociation between BCAT1 and various tumor types. Compared to BCAT2, BCAT1 exhibits more pronounced dysregulation across cancers and stronger clinical associations, playing a more direct role in tumor metabolic reprogramming, proliferation, and metastasis. Therefore, BCAT1 was selected for this pan-cancer study to systematically elucidate its expression patterns, prognostic relevance, and underlying mechanisms.

In our work, we performed a pan-cancer investigation of BCAT1 using The Cancer Genome Atlas (TCGA) and Genotype-Tissue Expression (GTEx) datasets. This study hypothesized that BCAT1 is upregulated across multiple cancer types and that its high expression correlates with poor patient prognosis. The primary endpoints were overall survival (OS) and disease-free survival (DFS), with further exploration of its associations with immune infiltration. We also incorporated an exploration of the possible molecular mechanism of BCAT1 in the pathophysiology and clinical prognosis of various cancers.

## 2. Materials and methods

### 2.1. Gene expression analysis

Gene expression and clinical data for pan-cancer analysis were obtained via the gene expression profiling interactive analysis 2 (GEPIA2) and tumor immune estimation resource 2 (TIMER2) web servers, which provide integrated, normalized, and batch-corrected data from TCGA and GTEx projects. Inclusion criteria were tumor and normal samples with complete expression profiles and clinical information; exclusion criteria were samples with incomplete data. A total of 12,554 samples across 33 cancer types were included.

The Human Protein Atlas (HPA) database (https://www.proteinatlas.org/) and GTEx database (https://www.gtexportal.org/home/) were used to obtain the mRNA expression profiles of normal tissues. In the “Gene_DE” module of TIMER2 online, we entered the gene BCAT1 and found the expression differences between the tumor and surrounding normal tissues for various malignancies or specific tumor subtypes from the TCGA dataset. Samples were dichotomized into high- and low-expression groups based on the median BCAT1 expression level within each cancer cohort. The expression analysis – box plots module of the GEPIA2 web server was used to get the result of the expression difference between normal tissues and the related tumor tissues of the GTEx database in the form of box plots, with the settings of *P* value = .01, log_2_ FC = 1, and the mode “Match TCGA normal and GTEx data” for a few tumors with limited normal tissues or without normal tissues. Expression data were normalized as transcripts per million (TPM) and batch-corrected using the ComBat method. Integration of TCGA and GTEx data employed the “Match TCGA normal and GTEx data” mode. Expression differences between tumor and normal tissues were assessed using unadjusted *P* values. For analyses involving multiple comparisons across cancer types, the Benjamini–Hochberg false discovery rate (FDR) correction was applied, with significance set at FDR < 0.05. The Cancer Cell Line Encyclopedia dataset provides cell line gene expression matrices for malignancies. Moreover, using the “Pathological Stage Plot” module of GEPIA2, we created violin plots of BCAT1 expression in various pathological phases of all TCGA tumors. BCAT1 expression levels in single cells were analyzed using the HPA datasets and a protein atlas (https://www.proteinatlas.org/).

### 2.2. Survival analysis

Patients within each cancer type were dichotomized into high- and low-expression groups based on the median BCAT1 expression level within that cohort. We obtained DFS and OS data of BCAT1 across all TCGA tumors using the Kaplan–Meier “Survival Map” module of GEPIA2. The hypothesis test employed the log-rank test, and GEPIA2’s “Survival Analysis” module also produced survival graphs. The Kaplan–Meier “Survival Map” and “Survival Analysis” modules of GEPIA2 were used to generate survival curves and log-rank *P* values. The shaded bands in the figures represent the 95% confidence intervals for the Kaplan–Meier survival probability estimates. For this initial pan-cancer screening, patients within each cancer type were dichotomized into high- and low-expression groups based on the median BCAT1 expression level within that cohort to ensure a standardized and comparable screening approach across all 33 cancer types. This survival analysis represents an initial exploratory screening to identify potential pan-cancer associations. Therefore, univariate Kaplan–Meier analysis with log-rank tests was employed without adjustment for clinical covariates.

### 2.3. Genetic alteration analysis

After logging into the cBioPortal online, we selected the “TCGA Pan Cancer Atlas Studies” under “Quick select” and typed “BCAT1” to search for information about BCAT1’s genetic alteration traits. The “Cancer Types Summary” module displayed the results of the copy number alteration, mutation type, and alteration frequency across all TCGA tumors. Through the “Mutations” module, the BCAT1 mutation site information can be viewed in either a schematic representation of the protein structure or a 3-dimensional model of the structure. This structure was selected due to its completeness, high quality, and direct correspondence to the UniProt P54687 sequence, providing a reliable framework for visualizing the protein’s functional domains and the potential spatial context of identified genetic alterations. Information on the variations in overall, disease-free, progression-free, and disease-free survival for TCGA cancer cases with or without BCAT1 genetic mutations was also obtained using the “Comparison” module. Additionally, Kaplan–Meier plots with log-rank *P* values were generated.

### 2.4. Immune infiltration analysis

With regard to all TCGA tumors, we investigated the relationship between BCAT1 expression and immune infiltrates using the “Immune-Gene” module of the TIMER2 web server. The CD8+ T cell immune system and cancer-associated fibroblasts were chosen. To estimate immune infiltration, Xenobiotic Chemical-Responsive Expression Loci, Microenvironment Cell Populations-counter, and Estimating the Proportion of Immune and Cancer cells (EPIC) algorithms were used. The purity-adjusted Spearman rank correlation test was applied to calculate partial correlation (cor) and *P* values between BCAT1 expression and immune cell infiltration levels for each cancer type. To account for multiple testing across cancer types and immune cell types, the Benjamini–Hochberg procedure was used to control the (FDR < 0.05). All reported correlations are based on purity-adjustedvalues unless otherwise stated. Scatter plots and heatmaps were generated to visualize significant associations (FDR < 0.05).

The association between BCAT1 expression and levels of immune cell infiltration (specifically CD8+ T cells and cancer-associated fibroblasts) was analyzed separately within each individual TCGA cancer type. For each cancer type, a purity-adjusted Spearman correlation was computed across all patient samples within that cancer cohort. The sample size for each cancer type was determined by the number of TCGA cases with available RNA-seq and clinical data. This sample size remained identical for all deconvolution algorithms applied to the same cohort, as each algorithm estimated infiltration scores from the same underlying gene expression matrix for every patient.

### 2.5. BCAT1-related gene enrichment analysis

First, we conducted a search on the STRING website using the terms “BCAT1” and “Homo sapiens” to identify single proteins. We then established the following key parameters: the minimal desired interaction score (0.150 for low confidence), the significance of network edges (“evidence”), the maximum interactors to display (at most 50 interactors), and the active interaction sources (“experiments”). Finally, experimentally determined BCAT1-binding proteins were retrieved.

Based on datasets of all TCGA normal and tumor tissues, the top 100 BCAT1-correlated genes were identified using GEPIA2’s “Similar Gene Detection” module with a Pearson |*R*| > 0.3 threshold across all TCGA tumors. For functional enrichment, the background gene set consisted of all protein-coding genes in the human genome. Gene ontology (GO) and Kyoto Encyclopedia of Genes and Genomes (KEGG) pathway analyses were performed using the R package clusterProfiler (version 4.0.5; Bioconductor). For enrichment analyses and multiple comparisons, statistical significance was defined by a Benjamini–Hochberg FDR < 0.05. For single comparisons where no multiple testing correction was applied (e.g., initial screening in GEPIA2), *P* < .05 was used as an exploratory threshold. Terms with gene counts ≥5 and FDR < 0.05 were considered significantly enriched. A conjugated gene Pearson correlation study of BCAT1 and a few other genes was also carried out using the “correlation analysis” module of GEPIA2. For the dot plot, log_2_ TPM was performed. There were indications of a *P* value as well as a correlation coefficient (*R*). Additionally, we obtained the heatmap data of the targeted genes using TIMER2’s “Gene_Corr” module, which includes the *P* value of Spearman grade correlation test after purity adjustment and partial correlation (cor).

The Search Tool for the Retrieval of Interacting Genes (STRING database; https://string-db.org/) was used to construct a protein–protein interaction network. Furthermore, we performed KEGG pathway analysis by combining the 2 sets of data. Briefly, we collected the data for the functional annotated map by uploading the gene list to the Database for Annotation, Visualization and Integrated Discovery database for annotation, visualization, and integration of findings with specific set identifiers (“OFFICIAL_GENE_SYMBOL”) and species (“*Homo sapiens*”). The enriched pathways were finally identified using the R packages “tidyr” and “ggplot2.” Furthermore, we performed GO enrichment analysis using the R package “cluster Profiler.” This study was conducted using the R language software [*R*-3.6.3, 64-bit] (https://www.r-project.org/). *P* < .05. for both tails was considered statistically significant.

## 3. Results

### 3.1. Gene expression analysis

**Figure 1.**
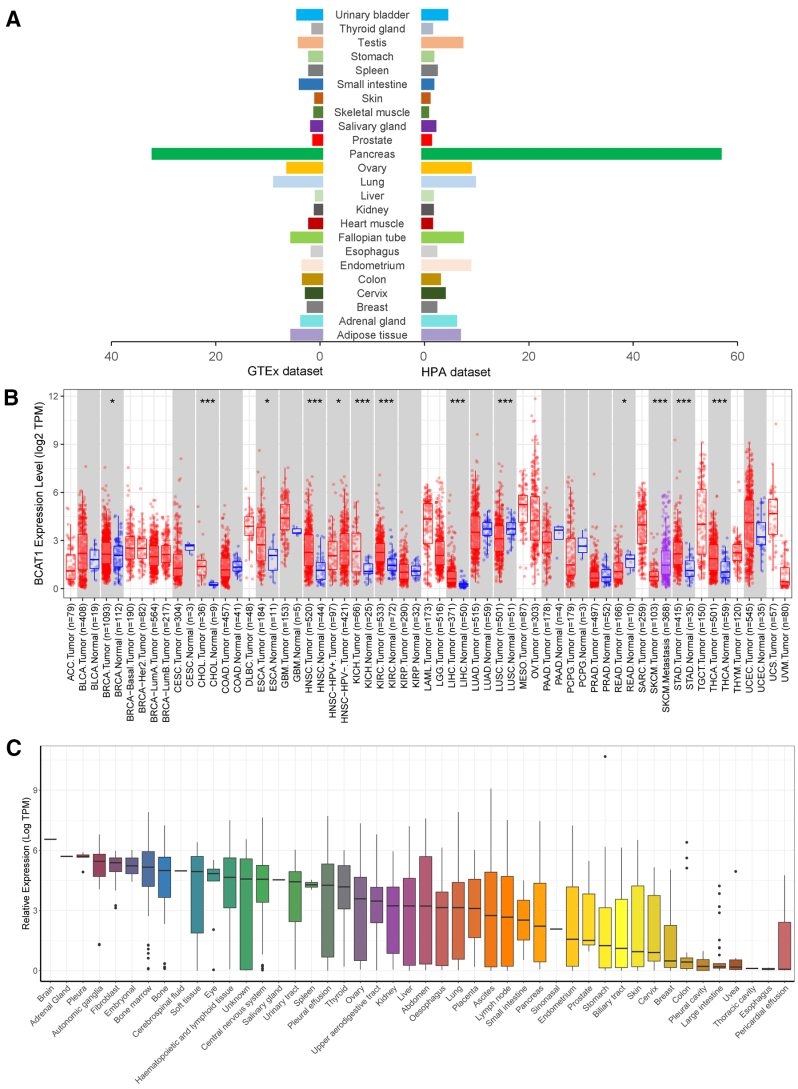
Expression level of BCAT1 in different normal and tumor tissues and pathological stages. (A) Expression level of BCAT1 in normal tissues in HPA datasets and GTEx datasets. (B) The expression status of the BCAT1 gene in different tumors or corresponding subtypes. * *P* < .05; ** *P* < .01; *** *P* < .001. (C) Expression levels of BCAT1 in cancer cell lines (CCLE datasets). BCAT1 = branched-chain aminotransferase 1, CCLE = Cancer Cell Line Encyclopedia, GTEx = Genotype-Tissue Expression, HPA = Human Protein Atlas, TPM = transcripts per million.

Analysis of the HPA and GTEx (Fig. 1A) datasets revealed higher expression of BCAT1 in the pancreas, lungs, and other normal tissues. We used TIMER2 to examine BCAT1 expression across different TCGA cancer types. As depicted in Figure 1B, the expression levels of BCAT1 in the tumor tissues of cholangiocarcinoma, head and neck squamous cell carcinoma, kidney chromophobe, kidney renal clear cell carcinoma, liver hepatocellular carcinoma, stomach adenocarcinoma, thyroid carcinoma (*P* < .001), breast invasive carcinoma, and esophageal carcinoma (*P* < .05) were higher than those in the corresponding control tissues. In contrast to tissue expression levels, as shown in Figure 1C, cell line expression analysis showed that BCAT1 was highly expressed in brain and adrenal gland cells. The expression of BCAT1 in normal and tumor tissues of lymphoid neoplasm diffuse large B-cell lymphoma and uterine carcinosarcoma (*P* < .05) was further assessed (Fig. S1A, Supplemental Digital Content 1). Based on the clinical proteomic tumor analysis consortium dataset, we also analyzed the expression levels of BCAT1 total protein in normal and primary tissues (Fig. S1B, Supplemental Digital Content 1). Additionally, we used the “Pathological Stage Plot” module of GEPIA2 to create violin plots of BCAT1 expression in each of the 4 pathological stages of all TCGA tumors (Fig. S1C, Supplemental Digital Content 1). For the box or violin plots, the modified expression data of log_2_(TPM + 1) were utilized.

### 3.2. Single-cell analysis

**Figure 2.**
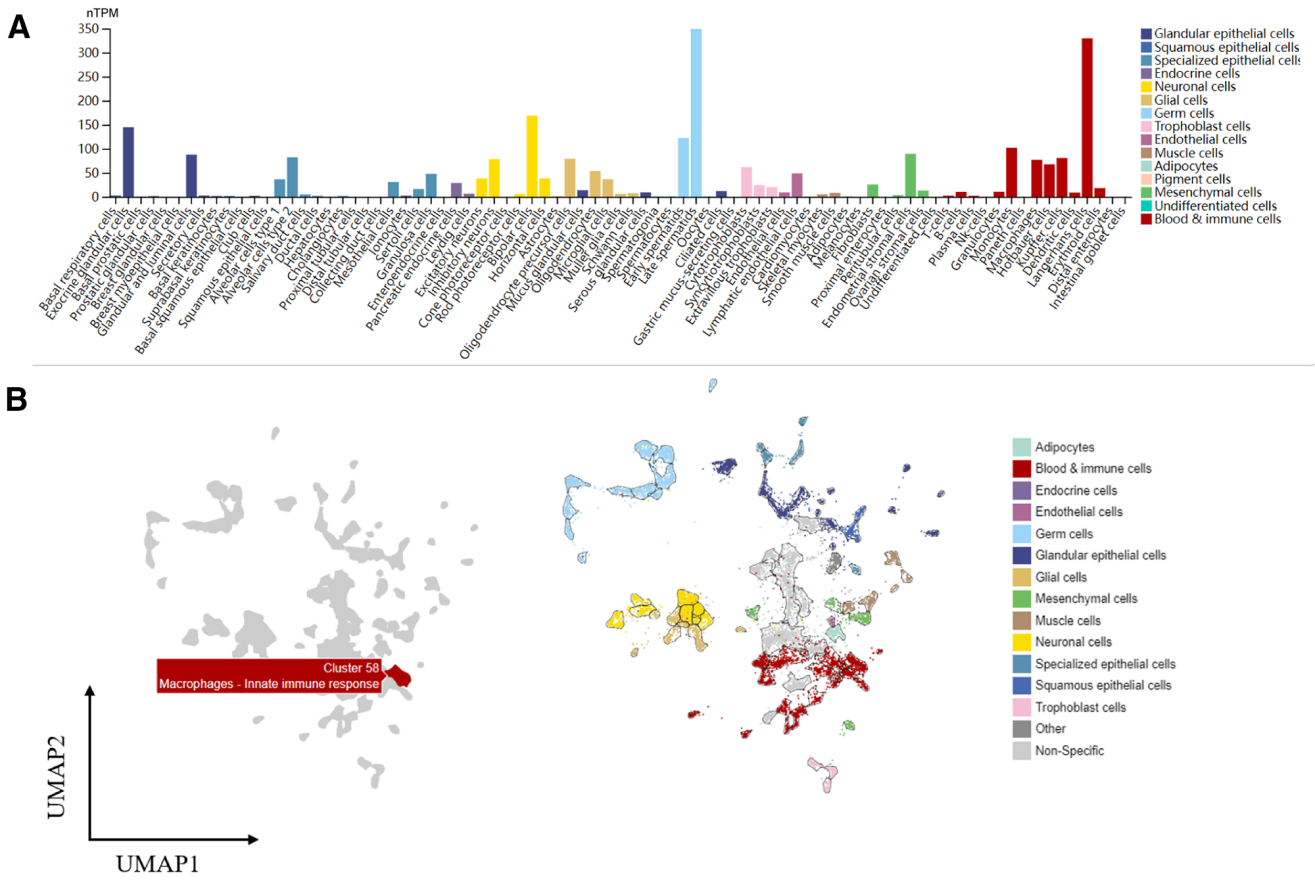
Analysis of BCAT1 in single-cell data. (A) BCAT1 expression levels in single cells (HPA datasets). BCAT1 is mainly expressed in blood and immune cells. (B) BCAT1 is part of cluster 58 macrophages – innate immune response with confidence 1. BCAT1 = branched-chain aminotransferase 1, HPA = Human Protein Atlas, TPM = transcripts per million, UMAP = Uniform Manifold Approximation and Projection.

Cell-specific expression of BCAT1 was revealed using single-cell analysis. Analysis of the HPA dataset revealed that BCAT1 was mainly expressed in blood and immune cells (Fig. 2A). Furthermore, correlation analysis between immune cell clustering and BCAT1 expression revealed that BCAT1 is a part of cluster 58 macrophages – innate immune response with confidence 1 (Fig. 2B).

### 3.3. Survival analysis

**Figure 3.**
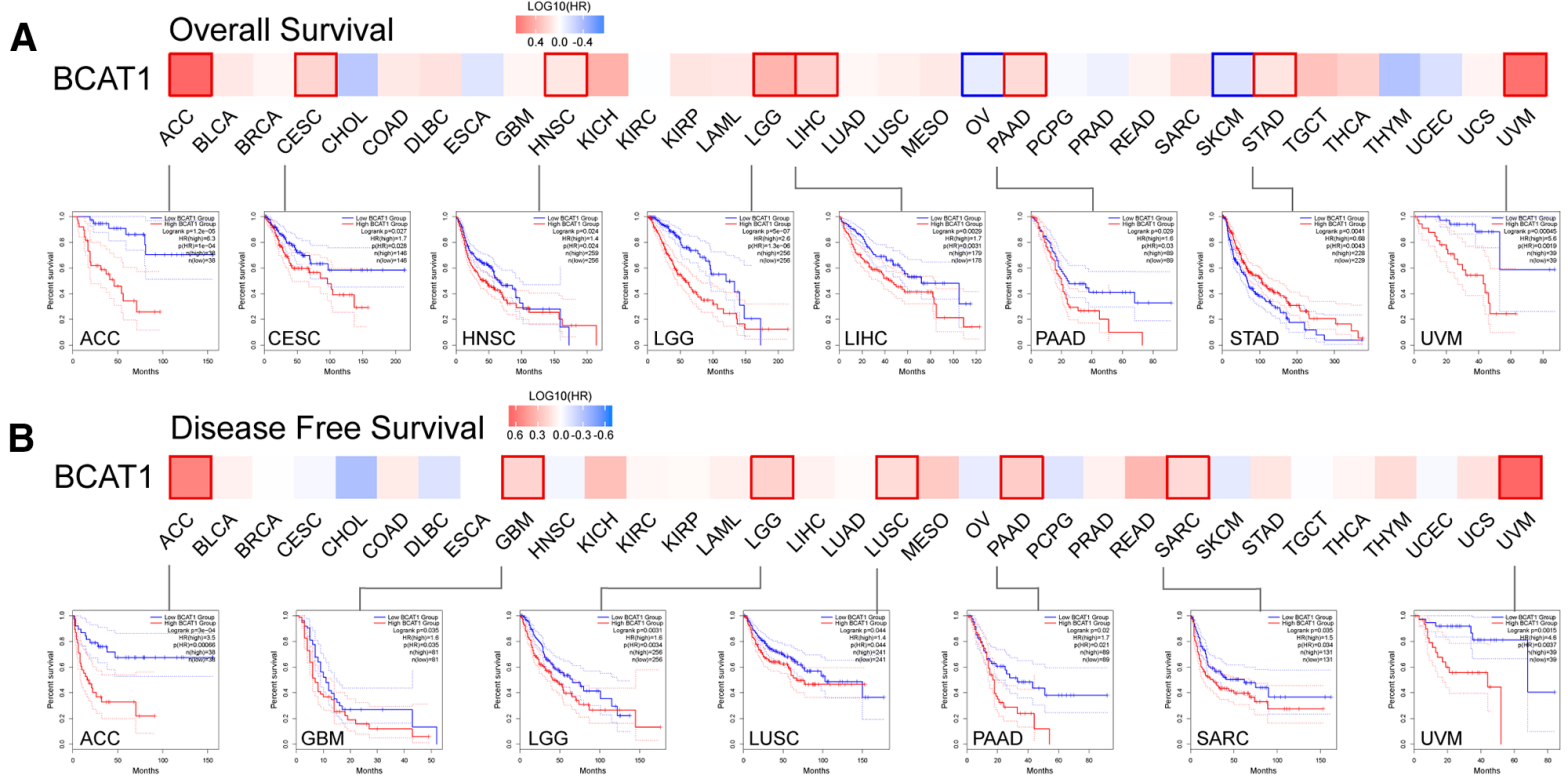
Correlation between BCAT1 gene expression and survival prognosis of cancers in TCGA. (A) We used the GEPIA2 tool to perform overall survival (OS) and (B) disease-free survival (DFS) analyses. Each square’s color represents the direction of the association (red: HR > 1; blue: HR < 1). Detailed Kaplan–Meier curves are shown for all cancer types where high BCAT1 expression was significantly associated with poorer survival and displayed the highest hazard ratios among significant results, serving as representative examples of the most robust negative prognostic associations identified. ACC = adrenocortical cancer, BCAT1 = branched-chain aminotransferase 1, BLCA = bladder urothelial carcinoma, BRCA = breast invasive carcinoma, CESC = cervical cancer, CHOL = cholangiocarcinoma, COAD = colon adenocarcinoma, DLBC = diffuse large B-cell lymphoma, ESCA = esophageal carcinoma, GBM = glioblastoma, GEPIA2 = gene expression profiling interactive analysis 2, HNSC = head and neck squamous cell carcinoma, HR = hazard ratio, KICH = kidney chromophobe, KIRC = kidney renal clear cell carcinoma, LAML = acute myeloid leukemia, LGG = lower grade glioma, LIHC = liver hepatocellular carcinoma, LUSC = lung squamous cell carcinoma, MESO = mesothelioma, OV = ovarian serous cystadenocarcinoma, PAAD = pancreatic adenocarcinoma, PCPG = pheochromocytoma and paraganglioma, PRAD = prostate adenocarcinoma, READ = rectum adenocarcinoma, SARC = sarcoma, SKCM = skin cutaneous melanoma, STAD = stomach adenocarcinoma, TCGA = The Cancer Genome Atlas, THCA = thyroid carcinoma, THYM = thymoma, UCEC = uterine corpus endometrial carcinoma, UCS = uterine carcinosarcoma, UVM = uveal melanoma.

Using TCGA datasets, we separated the cancer cases into high- and low-expression groups based on the expression levels of BCAT1 and examined the relationship between BCAT1 expression and the prognosis of patients with various cancers. The TCGA study revealed that there was a negative correlation between highly expressed BCAT1 and poor OS for malignancies of adrenocortical cancer (*P* < .001), cervical cancer (*P* = .028), head and neck squamous cell carcinoma (*P* = .024), and lower-grade glioma (*P* < .001) (Fig. 3A). High BCAT1 expression was correlated with poor prognosis in TCGA cases of adrenocortical cancer (*P* < .001), glioblastoma (*P* = .035), lower-grade glioma (*P* = .0034), and lung squamous cell carcinoma (*P* = .044), according to DFS analysis (Fig. 3B).

### 3.4. Genetic alteration analysis

**Figure 4.**
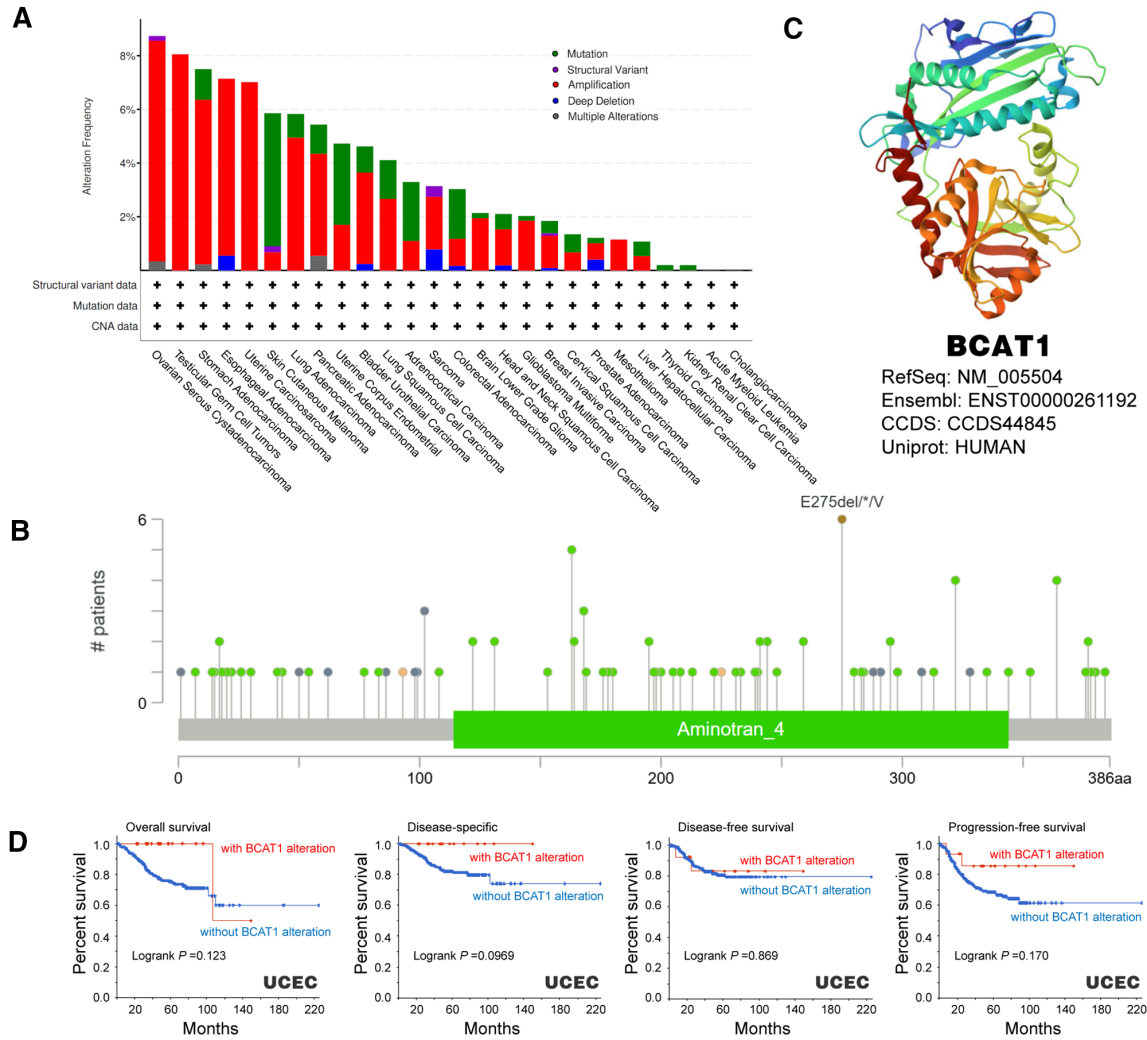
Mutation characteristics of BCAT1 in different tumors in TCGA. Using the cBioPortal tool, we examined the BCAT1 mutation characteristics for the TCGA cancers. The mutation type (A) and mutation site (B), along with the modification frequency, are shown. We presented BCAT1’s 3-dimensional structure (C). Using the cBioPortal tool, we also examined the possible relationship between the mutation status and the overall, disease-specific, disease-free, and progression-free survival of UCEC (D). BCAT1 = branched-chain aminotransferase 1, TCGA = The Cancer Genome Atlas, UCEC = uterine corpus endometrial carcinoma.

We investigated the genetic status of BCAT1 in several tumor samples from the TCGA cohort. Figure 4A shows that patients with cutaneous melanoma with the primary type “mutation” had the highest BCAT1 modification frequency. The “amplification” form of CNA was the most common in ovarian cancer cases, with an alteration frequency of >8% (Fig. 4A). It is worth mentioning that sarcoma cases with genetic changes exhibited a copy number loss of BCAT1 (Fig. 4A). Figure 4B shows the types, sites, and case numbers of the BCAT1 genetic mutations. The 3D structure of the BCAT1 protein is shown in Figure 4C. We also investigated the potential relationship between BCAT1 mutations and clinical survival in patients with various cancers. Although not statistically significant, the data in Figure 4D indicate that uterine corpus endometrial carcinoma cases with altered BCAT1 expression showed a tendency for better prognosis.

### 3.5. Immune infiltration analysis

**Figure 5.**
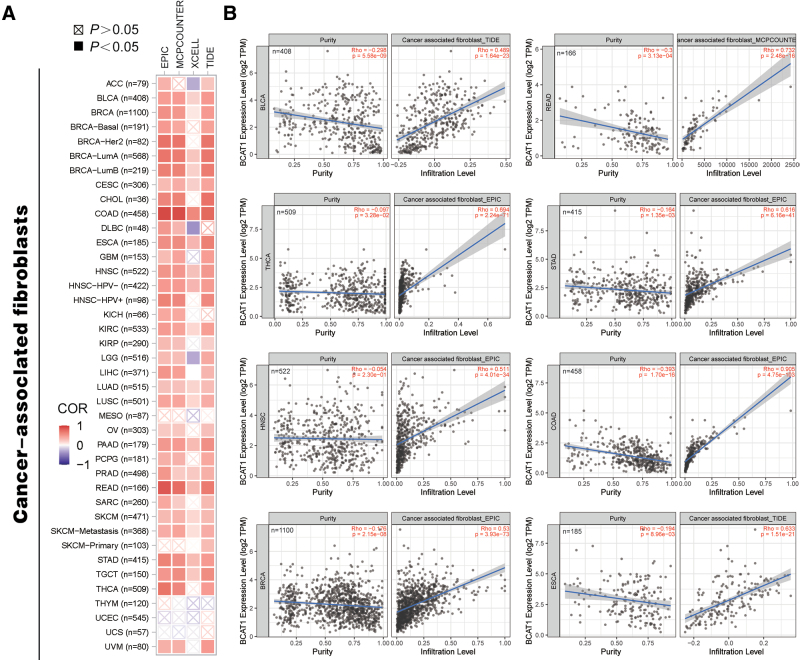
Analysis of the correlation between BCAT1 expression and immune infiltration of cancer-associated fibroblasts. The possible relationship between the BCAT1 gene expression level (A) and the infiltration level (B) of cancer-associated fibroblasts across all cancer types in TCGA was investigated using different algorithms. Representative scatter plots show the correlation between BCAT1 expression and cancer-associated fibroblast infiltration in selected cancer types. The sample size (n) for each cohort corresponds to panel A. BCAT1 = branched-chain aminotransferase 1, TCGA = The Cancer Genome Atlas.

As important elements of the tumor microenvironment, tumor-infiltrating immune cells are directly related to cancer initiation, development, and invasion.^[9]^ Cancer fibroblasts have been documented to play a role in regulating the activity of different immune cells that infiltrate tumors inside the stroma of the tumor microenvironment.^[10]^ Herein, we examined the potential connections between the degree of immune cell infiltration and BCAT1 gene expression in various TCGA cancer types using the Xenobiotic Chemical-Responsive Expression Loci, Microenvironment Cell Populations-counter, and EPIC algorithms. In addition, in TCGA tumors with almost all malignant tumors, especially colon adenocarcinoma, rectum adenocarcinoma, and cholangiocarcinoma, we observed a statistically positive association between BCAT1 expression and estimates of cancer-associated fibroblast infiltration values (Fig. 5A). To detail the most robust associations from the pan-cancer analysis, representative scatter plots are shown in Figure 5B. These plots were selected based on exhibiting the strongest positive correlation coefficients in Figure 5A. For example, based on the EPIC algorithm, the expression level of BCAT1 in breast invasive carcinoma positively correlated with the infiltration level of cancer-associated fibroblasts (cor = 0.53, *P* = 3.93E−73).

### 3.6. Enrichment analysis of BCAT1

**Figure 6.**
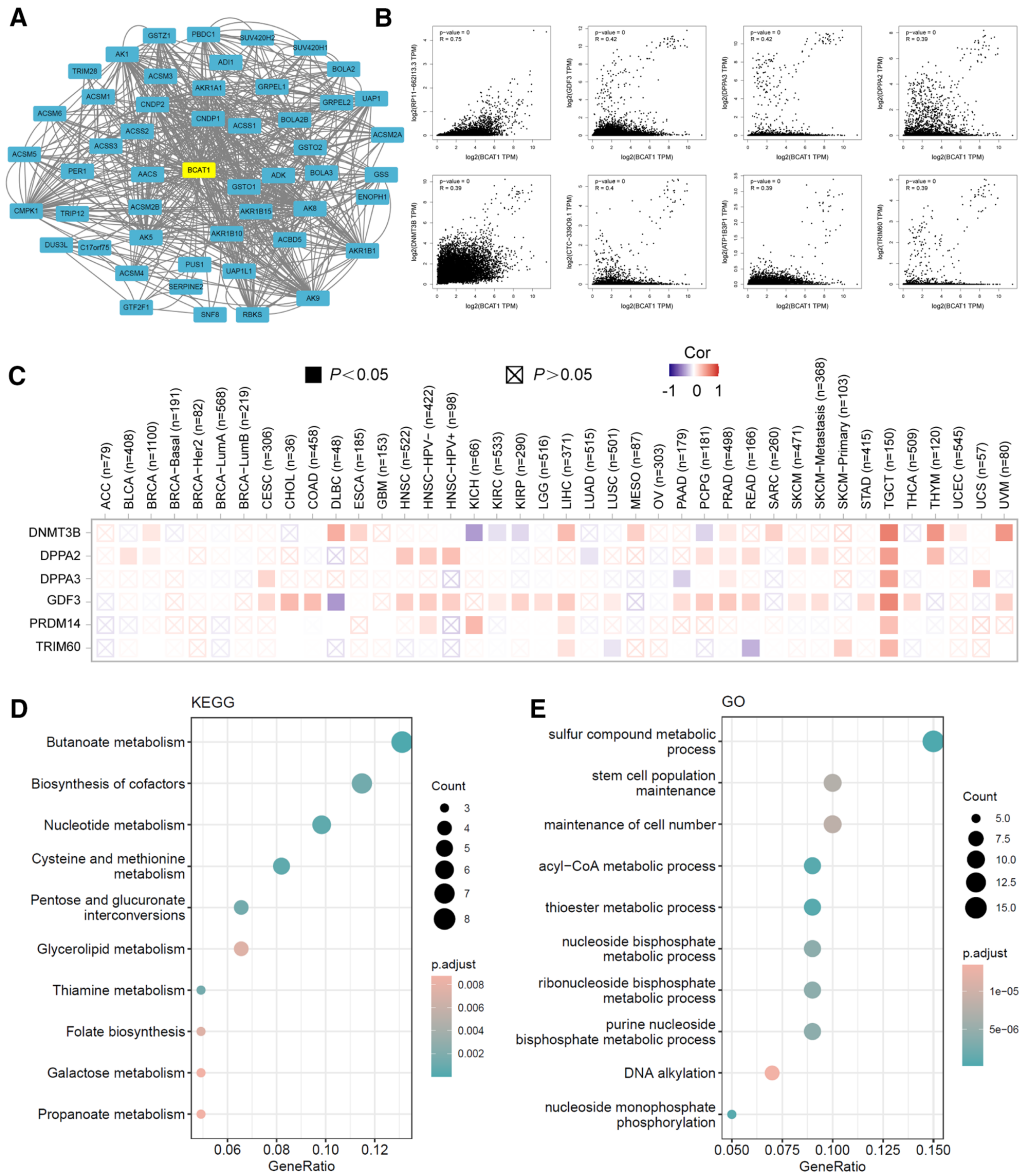
Gene enrichment analysis of BCAT1. (A) We 1st obtained the available experimentally determined BCAT1-binding proteins using the STRING tool. (B) Using the GEPIA2 approach, we also obtained the top 100 BCAT1-correlated genes in TCGA projects and analyzed the expression correlation between BCAT1 and selected targeting genes. (C) The corresponding heatmap data in the detailed cancer types are displayed. (D) Based on the BCAT1-binding and interacted genes, KEGG pathway analysis was performed. (E) The molecular function data in GO analysis was also shown. ACC = adrenocortical cancer, BCAT1 = branched-chain aminotransferase 1, BLCA = bladder urothelial carcinoma, BRCA = breast invasive carcinoma, CESC = cervical cancer, CHOL = cholangiocarcinoma, COAD = colon adenocarcinoma, DLBC = diffuse large B-cell lymphoma, ESCA = esophageal carcinoma, GBM = glioblastoma, GEPIA2 = gene expression profiling interactive analysis 2, GO = gene ontology, HNSC = head and neck squamous cell carcinoma, KEGG = Kyoto encyclopedia of genes and genomes, KICH = kidney chromophobe, KIRC = kidney renal clear cell carcinoma, LAML = acute myeloid leukemia, LGG = lower grade glioma, LIHC = liver hepatocellular carcinoma, LUSC = lung squamous cell carcinoma, MESO = mesothelioma, OV = ovarian serous cystadenocarcinoma, PAAD = pancreatic adenocarcinoma, PCPG = pheochromocytoma and paraganglioma, PRAD = prostate adenocarcinoma, READ = rectum adenocarcinoma, SARC = sarcoma, SKCM = skin cutaneous melanoma, STAD = stomach adenocarcinoma, STRING = Search Tool for the Retrieval of Interacting Genes, TCGA = The Cancer Genome Atlas, THCA = thyroid carcinoma, THYM = thymoma, UCEC = uterine corpus endometrial carcinoma, UCS = uterine carcinosarcoma, UVM = uveal melanoma.

In order to conduct a series of pathway enrichment analyses, we tried to filter out the targeting BCAT1-binding proteins and the genes that were associated with BCAT1 expression in order to learn more about the molecular mechanism of the BCAT1 gene in carcinogenesis. We identified 50 BCAT1-binding proteins, all of which were supported by experimental data, using the STRING program. The network of these proteins is shown in Figure 6A. The top 100 genes linked to BCAT1 expression were identified by combining all TCGA tumor expression data using the GEPIA2 algorithm. The degree of BCAT1 expression was strongly linked with growth differentiation factor 3 (*R* = 0.42) and developmental pluripotency associated 2 (*R* = 0.39) expression (Fig. 6B). The heatmap data also revealed a positive connection between BCAT1 and the aforementioned genes in the majority of specified cancer types (Fig. 6C). To conduct KEGG and GO enrichment analyses, we integrated these 2 datasets. The KEGG data in Figure 6D suggest that “butanoate metabolism” and “biosynthesis of cofactors” may be involved in the effect of BCAT1 on tumor pathogenesis. The majority of these genes were associated with metabolic processes, including the metabolism of sulfur compounds, acyl-CoA, and other metabolic pathways, according to the GO enrichment analysis results (Fig. 6E).

## 4. Discussion

Metabolic reprogramming may be necessary to maintain abnormal cell proliferation, especially when nutrients are restricted.^[11,12]^ Additionally, metabolites can transmit signals that coordinate operations such as gene expression and nutrient uptake. Disrupting these processes can prevent both cell proliferation and tumor formation in cancer cells, which must modify their metabolism to enable biomass creation, adenosine triphosphate synthesis, and redox state maintenance.^[13]^ Understanding how cells consume and detect nutrients offers a chance to take advantage of the metabolic alterations observed in cancer and enhance patient care. Although it is well known that many cancer types exhibit dysregulated glucose metabolism, an increased requirement for amino acids must also be satisfied to maintain cell proliferation and cancer development.^[14]^ One class of amino acids, the metabolism of which has been linked to particular cancer characteristics. Alterations in BCAA metabolism could affect both intrinsic cancer features of the cell and systemic metabolic abnormalities linked to certain malignancies. As a result, BCAA metabolism can affect various cancer characteristics and act as a pathological disease marker.

A recent rush of research on BCAA metabolism in cancer has concentrated primarily on BCAT1, whose expression is altered in various malignancies and often corresponds to poor outcomes.^[7,15,16]^ Shu et al reported that BCAT1 is overexpressed in gastric cancer patients and is linked to a worse survival rate.^[17]^ Our results showed that high expression of BCAT1 in digestive system tumors, such as stomach adenocarcinoma, liver hepatocellular carcinoma, and pancreatic adenocarcinoma, was positively associated with poor OS (Fig. 3A). This report revealed that BCAT1 may function as an oncogene by promoting proliferation, invasion, and angiogenesis via the PI3K/AKT/mTOR pathway. Mao et al demonstrated that BCAT1 was overexpressed not only in metastatic lung cancer cells but also in metastatic tissues at the protein level.^[18]^ These findings indicate that BCAT1 plays a significant role in lung cancer cell metastasis and may establish a unique target for antimetastatic therapy. Similarly, our results also showed that high expression of BCAT1 in lung squamous cell carcinoma predicted shorter disease-free survival (Fig. 3B), suggesting that high expression of BCAT1 promotes tumor recurrence. Xu et al reported that the expression level of BCAT1 in hepatocellular carcinoma tissues was substantially correlated with tumor number, tumor-node-metastasis stage, and tumor differentiation.^[19]^ Zhang et al demonstrated that BCAT1 is overexpressed in malignant melanoma tissues in both mice and humans. In addition, BCAT1 knockdown lowered oxidative phosphorylation and inhibited the proliferation and migration of melanoma cells.^[20]^ This is an indication that BCAT1 could be regarded as a prospective therapeutic target for the treatment of malignant melanomas, according to their results taken together. Safavi et al reported novel focal deletions of BCAT1 in adult lymphoblastic leukemia, and the prognosis for adult cases of this disease is extremely poor.^[21]^

Besides contributing to tumor formation, BCAT1 may also be a useful molecule for monitoring the effectiveness of immunotherapy. Peng et al reported that BCAT1 is associated with immune infiltration and could also affect the tumor microenvironment, which may contribute to the development and progression of oral squamous cell carcinomas.^[22]^ Thus, BCAT1 may act as a prospective target for oral squamous cell carcinomas immunotherapy interventions as well as a prognostic biomarker of survival outcomes. Chen et al demonstrated that the control of the tumor immunological microenvironment and the activation of numerous oncological pathways in pancreatic cancer were both dramatically affected by metabolism-related genes, which include BCAT1.^[23]^

The exact mechanism by which BCAT1 expression stimulates tumor growth is unknown, but it probably varies from tumor to tumor. During the onset and progression of gastric cancer, BCAT1 may stimulate the PI3K/AKT/mTOR pathway, which encourages angiogenesis, invasion, and proliferation.^[17]^ Experiments have shown that BCAT1 is necessary for the growth and development of breast cancer and that it plays a role in the metastatic spread of breast cancer cells when the BCAT gene is knocked out.^[24]^ Through the insulin-like growth factor 1 pathway, BCAT1 regulates triple-negative breast cancer cell invasion, migration, and proliferation. According to Mao et al, BCAT1 regulates the growth and metastasis of cancer cells in the human body and is highly expressed in metastatic lung cancer cells.^[18]^ Through a thorough analysis of numerous publications on lung cancer, we discovered that BCAT1 stimulates the malignant development of non-small cell lung cancer through a variety of induction routes.^[25]^

This study has several limitations. First, as a bioinformatics analysis relying on public sequencing data from TCGA and GTEx (accessed via GEPIA2 and TIMER2), it is subject to platform heterogeneity, batch effects, and variations in sample processing across different cohorts. Second, unmeasured confounding factors may influence the observed associations, as our survival analyses were unadjusted for clinical covariates. Third, the survival analyses presented, while revealing significant univariate associations in several cancer types, are exploratory and unadjusted for clinical covariates. Future research should prioritize validating these findings in independent cohorts and performing multivariable Cox regression analyses adjusted for key clinical-pathological factors to determine the independent prognostic value of BCAT1. Finally, our findings are correlative and do not establish causality. Future work should include experimental validation using in vitro and in vivo models to elucidate the mechanistic roles of BCAT1 in specific cancer types. Prospective clinical cohorts and single-cell multi-omics approaches are also needed to validate BCAT1 as a biomarker and explore its utility in predicting immunotherapy response.

## 5. Conclusion

In conclusion, the evidence clearly suggests that BCAT1 plays a significant role in a variety of cancer types, most likely through a number of distinct processes that are specific to each type of cancer. Here, our initial pan-cancer investigations of BCAT1 revealed statistical relationships between BCAT1 expression, clinical prognosis, and immune cell infiltration, which help to clarify the function of BCAT1 in carcinogenesis from the standpoint of clinical tumor samples.

## Acknowledgments

The authors thank all those who participated in this study.

## Author contributions

**Conceptualization:** Ming Sun.

**Data curation:** Yize Wei.

**Investigation:** Yize Wei.

**Methodology:** Mingjie Dong.

**Software:** Congrong Gao.

**Validation:** Binbin Wang, Congrong Gao, Hui Yue.

**Visualization:** Hui Yue.

**Writing – original draft:** Binbin Wang.

**Writing – review & editing:** Ming Sun.


