# Supplementary figures and images for: A pan-cancer analysis of the oncogenic role of branched-chain aminotransferase 1 (BCAT1) in human tumors
Source: Medicine (Baltimore). 2026 Jul 31;105(31):e50004. doi: 10.1097/MD.0000000000050004 (PMC13433127; doi:10.1097/MD.0000000000050004)

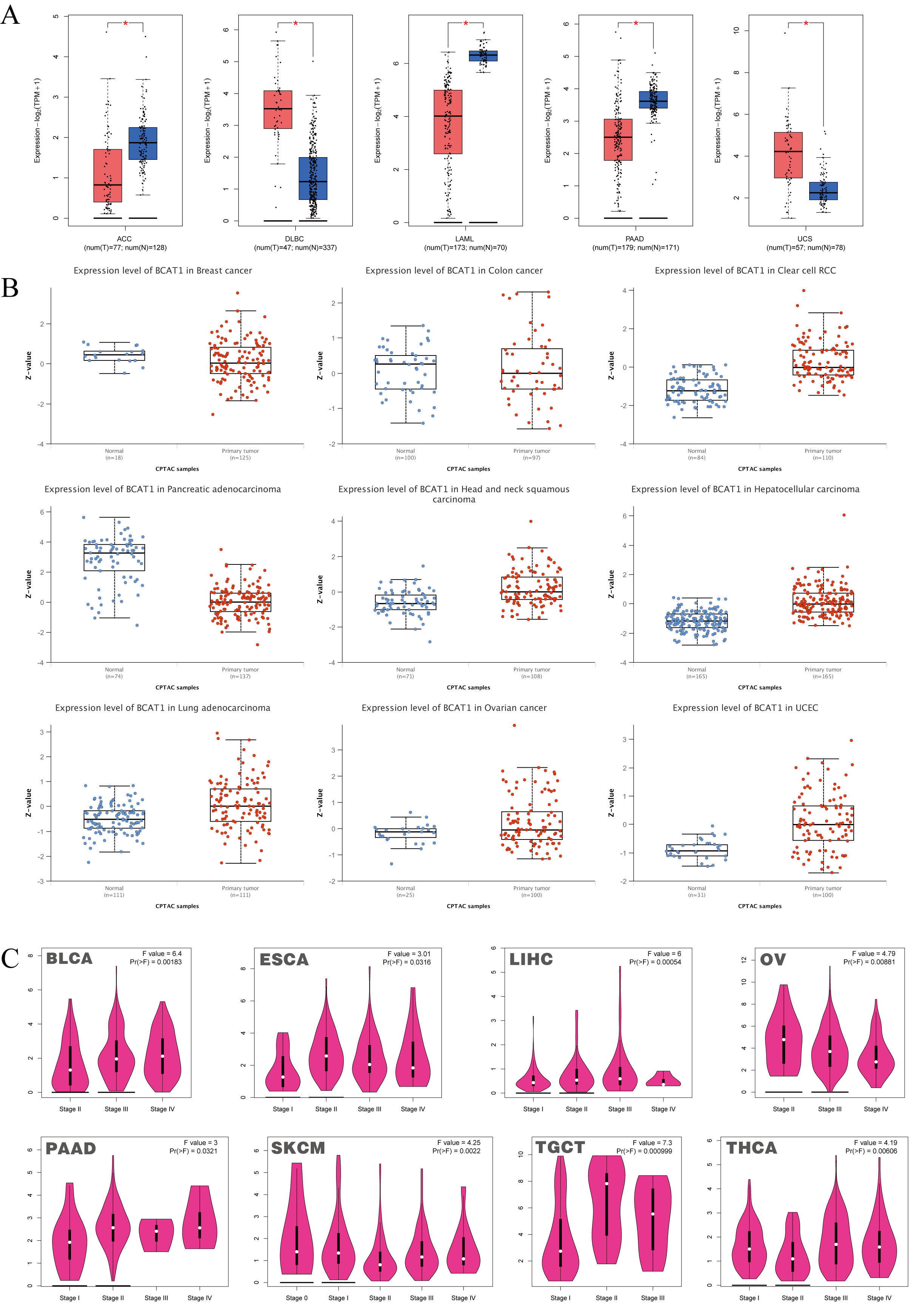

Supplement: Supplementary file 1 [file medi-105-e50004-s001.tif]
